# To predict sufentanil requirement for postoperative pain control using a real-time method: A prospective observational cohort study

**DOI:** 10.1097/MD.0000000000003915

**Published:** 2016-06-24

**Authors:** Yuhao Zhang, Guangyou Duan, Shanna Guo, Ying Ying, Penghao Huang, Mi Zhang, Ningbo Li, Xianwei Zhang

**Affiliations:** a Department of Anesthesiology, Tongji Hospital, Tongji Medical College, Huazhong University of Science and Technology, Wuhan, China; b Department of Anesthesiology, Xinqiao Hospital, Third Military Medical University, Chongqing, China.

**Keywords:** opioid, pain measurement, postoperative pain, prediction, sufentanil

## Abstract

Preoperative identification of individual sensitivity to opioid analgesics could improve the quality of postoperative analgesia. We explored the feasibility and utility of a real-time assessment of sufentanil sensitivity in predicting postoperative analgesic requirement.

Our primary study included 111 patients who underwent measurements of pressure and quantitative pricking pain thresholds before and 5 minutes after sufentanil infusion. Pain intensity was assessed during the first 24-hour postsurgery, and patients who reported inadequate levels of analgesia were excluded from the study. The sufentanil requirement for patient-controlled analgesia was recorded, and a subsequent exploratory study of 20 patients facilitated the interpretation of the primary study results. In the primary study, experimental pain thresholds increased (*P* < 0.001) 5 minutes after sufentanil infusion, and the percent change in pricking pain threshold was positively associated with sufentanil requirement at 12 and 24 hours after surgery (*β* = 0.318, *P* = 0.001; and *β* = 0.335, *P* = 0.001). A receiver-operating characteristic curve analysis showed that patients with a change in pricking pain threshold >188% were >50% likely to require more sufentanil for postoperative pain control. In the exploratory study, experimental pain thresholds significantly decreased after the operation (*P* < 0.001), and we observed a positive correlation (*P* < 0.001) between the percent change in pricking pain threshold before and after surgery. Preoperative detection of individual sensitivity to sufentanil via the above described real-time method was effective in predicting postoperative sufentanil requirement. Thus, percent change in pricking pain threshold might be a feasible predictive marker of postoperative analgesia requirement.

## 1 Introduction

Despite the development of numerous approaches in managing acute postoperative pain, the issue remains a relevant clinical challenge.^[1,2]^ Opioids are the most commonly used and effective analgesics for the relief of postoperative pain; however, failure to achieve appropriate dosing because of interindividual variation in opioid sensitivity reduces the quality of postoperative pain treatment.^[1,3,4]^ Several recent studies have described methods for predicting opioid analgesic requirements for postoperative pain control, such as opioid-related genotype screening^[5–7]^ and measurement of the papillary reflex after patients awaken from general anesthesia.^[8]^ However, some potential limitations impede the clinical application of these methods. For example, genotype screening is time-consuming and may need further validation, whereas measurement of the pupillary reflex can be confounded by other medications such as anticholinergic agents. Therefore, it is still necessary to explore definitive, rapid, and low-cost strategies for predicting the necessity of postoperative opioid analgesic treatment.

Recently, we investigated individual sensitivity to fentanyl via a real-time method that measured experimental pain before anesthesia induction in the operating room.^[9]^ Other studies have also used experimental pain measures to determine opioid sensitivity in patients with chronic pain or healthy volunteers.^[10–12]^ However, it is still unclear whether such real-time measures can predict opioid dose requirement. Therefore, the present study assessed the potential of this method to predict postoperative analgesic requirement using sufentanil, an opioid commonly used in China for anesthesia induction and postoperative pain management.^[13,14]^

Unexpectedly, our study demonstrated a correlation between higher preoperative sufentanil sensitivity and increased analgesic requirement for postoperative pain control. This counterintuitive result requires further investigation. Patient exposure to opioids during preoperative and intraoperative periods is known to cause opioid-induced hyperalgesia (OIH), which is one of the primary causes of increased analgesic requirement.^[1,15,16]^ Thus, we speculated that the association between OIH level and sensitivity to sufentanil may have contributed to the findings of our primary study. We therefore performed an exploratory study to test our hypothesis and to aid in the interpretation of our results.

## 2 Methods

### 2.1 Patients

The study was approved by the Huazhong University of Science and Technology Tongji Hospital Ethics Committee (approved ID: 2012–098), and written informed consent was obtained from all patients before their enrollment.

**Figure 1:**
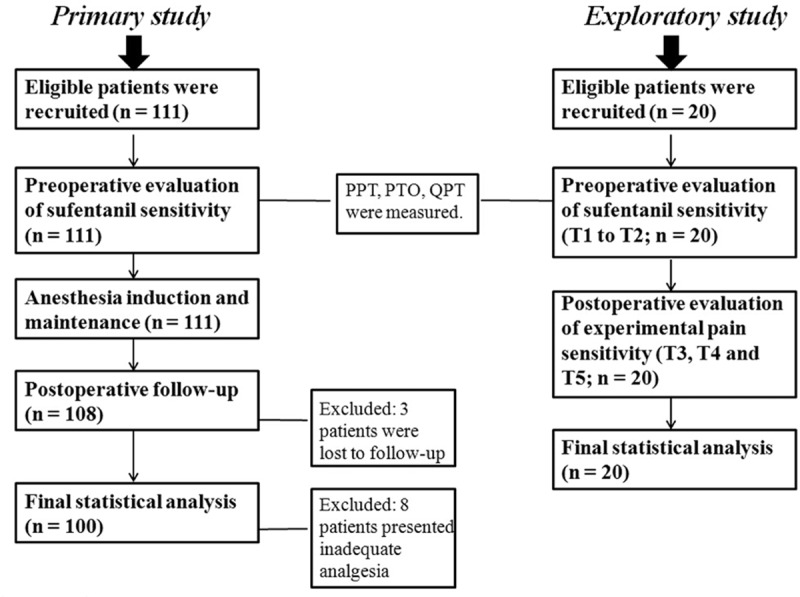
Flow diagram of primary and exploratory sample enrollment. PPT = pressure pain threshold, PTO = pressure pain tolerance, QPT = quantitative pricking pain threshold.

As shown in Fig. 1, 111 female patients (age 20–65 years) scheduled for gynecologic surgery under general anesthesia were recruited in the primary sample. The inclusion criteria were as follows: (1) voluntarily received postoperative patient-controlled analgesia (PCA); (2) right-hand dominance; and (3) grouping based on the American Society of Anesthesiologists physical status I to II. Exclusion criteria were as follows: (1) known history of chronic pain or use of any analgesic medication over the prior 4 weeks; (2) presence of dermatitis or damaged, red, or swelling skin at the selected test sites; and (3) preoxygenation pulse oxygen saturation that could not be maintained at 90% or above before induction of anesthesia. A subsequent exploratory sample of 20 patients, with the same inclusion and exclusion criteria as the primary sample, was recruited to aid in the interpretation of results from the primary study.

### 2.2 Preoperative management and preparation

Investigators screened potential study participants in the gynecological ward 1 day before the patients’ scheduled operations. Standardized instructions were given for the study procedure, and mechanical pain sensitivity tests were performed using the left forearm to familiarize subjects with the testing procedure at the outset of each testing session. After patients entered the operating room on the day of their scheduled surgeries, electrocardiogram, blood pressure, heart rate (HR), and pulse oxygen saturation (SpO_2_) were monitored. All patients were preoxygenated with 6 L/min oxygen via a facemask for 3 minutes before the study onset.^[17]^

### 2.3 Individual sensitivity to sufentanil before anesthesia induction

Based on the experience of our previous study^[9]^ and a pilot study aimed at detecting individual sensitivity to sufentanil, we used a reduced sufentanil dose of 0.4 μg/kg to avoid respiratory depression and excessive sedation. The 0.4 μg/kg sufentanil dose was diluted to 10 mL and then infused within 2 minutes. During the procedure, continuous oxygen was given to the patients via a facemask, and the test was stopped when SpO_2_ was <90%. Mechanical pain sensitivity tests were used to measure the analgesic effect. Before (T1) and 5 minutes after (T2) sufentanil infusion, mechanical pain sensitivity was measured. HR, SpO_2_, and blood pressure (mean arterial pressure [MAP]) were also recorded.

Similar to previous studies,^[18,19]^ a hand-held electronic mechanical algometer (YISIDADS2; Hong Kong, China) was used to measure mechanical pain sensitivity as defined by the pressure pain threshold (PPT) and pressure pain tolerance (PTO) with 0.1 cm^2^ probes. We also measured the quantizing pricking pain threshold (QPT) using the mechanical algometer and a 0.01 cm^2^ probe.

**Figure 2:**
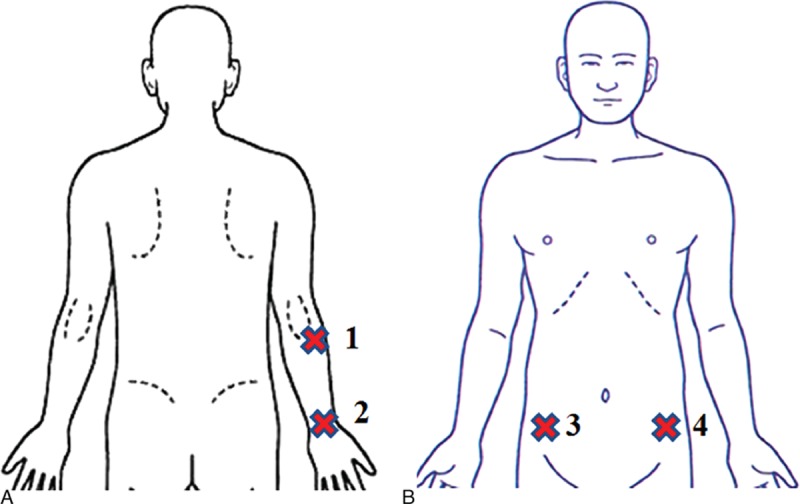
A, Test locations 1 and 2 (each marked with an X) on the right forearm. B, Test locations 3 and4 on the anterior superior iliac spine.

Two test locations were selected on the right forearm for the stimulus-evoked pain tests (Fig. 2A).^[18,20]^ The investigator applied the algometer for the pressure pain test at location 1, which corresponded to the lateral brachioradialis of the elbow joint. For the pricking pain test at location 2, the investigator applied the algometer to the midpoint of the medial and lateral borders of the wrist. A standardized procedure was used for all subjects, who were asked to say “pain” when they started to feel pain (PPT or QPT) during stimulation and “okay” when the pain became intolerable (PTO). This procedure was repeated 1 minute later, and the average of the 2 measurements was calculated.

### 2.4 Anesthetic technique

Standardized general anesthesia was administered to all patients after the testing procedure using 0.05 mg/kg midazolam, 2 mg/kg propofol, 0.3 μg/kg sufentanil, and 0.6 mg/kg rocuronium. Anesthesia was maintained with a combined intravenous and inhalation approach: inhalation of 1% to 2% sevoflurane, infusion of remifentanil (0.2 μg/kg/min) and propofol (6–10 mg/kg/h), and intravenous boluses of 0.2 mg/kg rocuronium. The depth of anesthesia was monitored using a Narcotrend instrument (Monitor Technik, Bad Bramstedt, Germany), and the Narcotrend index was controlled within the range of 20 to 46.^[21]^

### 2.5 Assessment and management of postoperative pain

Standard analgesia according to protocols of the acute pain service (APS) in our hospital, including preoperative analgesia and postoperative intravenous PCA, was provided for all patients. For postoperative pain management and prevention of postoperative nausea and vomiting (PONV), 40 mg of parecoxib sodium and 2 mg of tropisetron hydrochloride were administered 15 minutes before incision. PCA was started immediately after surgery using a controlled infusion pump containing sufentanil at 100 μg/100 mL.The pump was programmed to use a loading dose of 2 mL, background infusion at 1.5 mL/h, PCA dose of 1 mL, and lockout period of 10 minutes. Patients were monitored for 30 minutes in the postanesthesia care unit.

In the first 24 hour after surgery, all patients received PCA and were followed up by the investigator. When a patient reported inadequate analgesia (defined as presenting VAS >40—even after timely adjustment of the PCA pump), additional medications (e.g., flurbiprofen axetil, parecoxib sodium, and tramadol) were provided; however, it should be noted that such patients were excluded from the final analysis. Sufentanil consumption at 12 and 24 hours after surgery was recorded from the PCA pump and analyzed as μg/kg body weight. Adverse effects such as respiratory depression, pruritus, and PONV were also recorded.

### 2.6 Exploratory study

An additional 20 patients were recruited to validate the occurrence of OIH and its possible association with sufentanil sensitivity. In this exploratory sample, OIH was evaluated using mechanical measurement methods.^[22,23]^ As in the primary sample, individual sensitivity to sufentanil was evaluated using pressure pain (PPT, PTO) and pricking pain (QPT) measurements before anesthesia induction. Similar to previous studies, the sites near the surgical incision were considered to evaluate postoperative hyperpathia (Fig. 2B).^[24,25]^ In the exploratory study, test location 3 (right side of the anterior superior iliac spine) was used for the PPT and PTO, whereas test location 4 (left side of the anterior superior iliac spine) was selected for the QPT. Patients in the exploratory study underwent additional PPT, PTO, and QPT measurements at 8 hours (T3), 16 hours (T4), and 24 hours (T5) after the operation.

### 2.7 Statistical analysis

In the primary study, a maximum of 9 independent variables were included in the final multivariate linear stepwise regression analysis. Therefore, a sample size of 100 was considered sufficient for the study. For the exploratory study, a minimum of 10 individuals was required for a repeated analysis of variance (ANOVA); this figure was based on an estimated 20% change in postoperative QPT at a significance level of 0.05 and power of 0.9 using the sample size calculation software tool, PASS version 11.0 (NCSS, Kayesville, UT).

All variables were summarized using standard descriptive statistics, such as the mean, standard deviation (SD), and frequency. Paired-samples *t* test was used to compare PPT, PTO, QPT, HR, MAP, and SpO_2_ at T1 and T2. Individual sufentanil sensitivity was calculated as the percent change in PPT, PTO, and QPT after sufentanil infusion at T2 versus T1. A Pearson correlation analysis was used to compare the 12 and 24-hour PCA sufentanil requirement with preoperative data including age; body max index (BMI); basal pain sensitivity including PPT, PTO, and QPT at T1; the percent change of PPT, PTO, and QPT; and the duration of surgery. Then, multivariate linear stepwise regression models were used to evaluate the preoperative data as predictors of the postoperative PCA sufentanil requirement at 12 and 24-hours.

In the exploratory study, a repeated ANOVA was used to compare experimental pain sensitivity at different time points, and least significance difference (LSD) testing was used for multiple comparisons. The percent change in PPT, PTO, and QPT at preoperative T2 and postoperative T3, T4, and T5 versus T1 was calculated. A Pearson correlation analysis was used to compare the preoperative and postoperative percent change in PPT, PTO, and QPT.

We also calculated the unilateral 90% normal reference value for postoperative sufentanil consumption and defined patients with postoperative sufentanil consumption above this value as “excess requirement patients.” An exploratory receiver-operating characteristic (ROC) analysis was performed to determine optimal cut-off values for excess requirement, and the area under the curve (AUC) was calculated to assess the overall predictive accuracy of the percent change in QPT. Statistical analyses were performed using SPSS for Windows version 17.0 (SPSS Inc., Chicago, IL); a 2-tailed *P* < 0.05was considered statistically significant.

## 3 Results

### 3.1 General results

In total, 131 female patients were recruited into the primary and exploratory samples (Fig. 1). During sufentanil sensitivity testing, none of the patients presented SpO_2_ <90%, and 3 patients chose to withdraw from the primary study. To ensure that postoperative sufentanil consumption was similar for all patients, and to avoid the confounding effect of additional medications, 8 patients were excluded for inadequate postoperative analgesia. However, all patients in the exploratory study completed the study procedure. Therefore, in the final analysis, 100 patients were included in the primary study and 20 patients were included in the exploratory study. Demographic data are shown in Table 1.

**Table 1:**
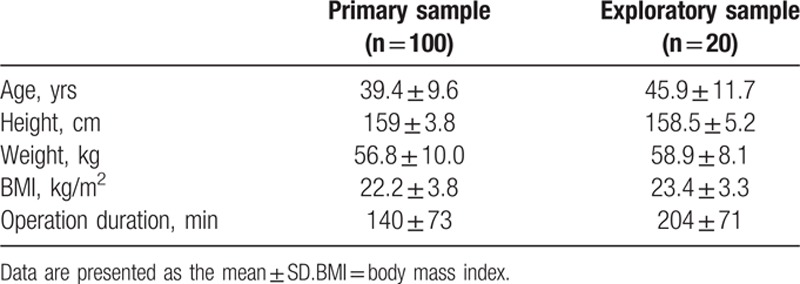
Demographic data of primary and exploratory samples.

### 3.2 Preoperative data and analgesic outcomes in the primary study

In the primary study, PPT, PTO, and QPT significantly increased after sufentanil infusion (*P* < 0.001) and there was no significant change in HR, MAP, and SpO_2_ at 5 minutes after infusion (*P* > 0.05; Table 2). Based on these results, we calculated individual sufentanil sensitivity as the percent change in PPT, PTO, and QPT. Sufentanil requirements within 12 hours after surgery averaged 21.4 ± 6.8 μg or 0.374 ± 0.092 μg/kg; at 24 hours after surgery, these requirements were 41.4 ± 13.0 μg and 0.722 ± 0.170 μg/kg. No respiratory depression or pruritus was observed; PONV was noted in 8 patients (8%).

**Table 2:**
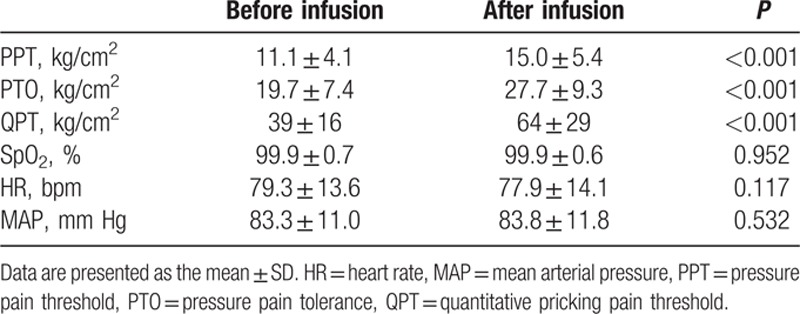
Measurements before and after sufentanilinfusion in the primary study.

### 3.3 Predictive model

As shown in Table 3, the sufentanil requirement within 12 and 24 hours after surgery was positively correlated with the percent change in QPT (*P* = 0.001 at both time points) and negatively correlated with preoperative basal QPT (*P* = 0.002, 12 hours; *P* = 0.005, 24 hours). Thus, the final predictive model was developed through a multiple regression analysis of the postoperative sufentanil requirement, and basal QPT and percent change in QPT were included. Collinearity diagnostics for basal QPT and percent change in QPT showed that the variance inflation factor (VIF) was 1.229, indicating that these 2 factors were independent of each other.

**Table 3:**
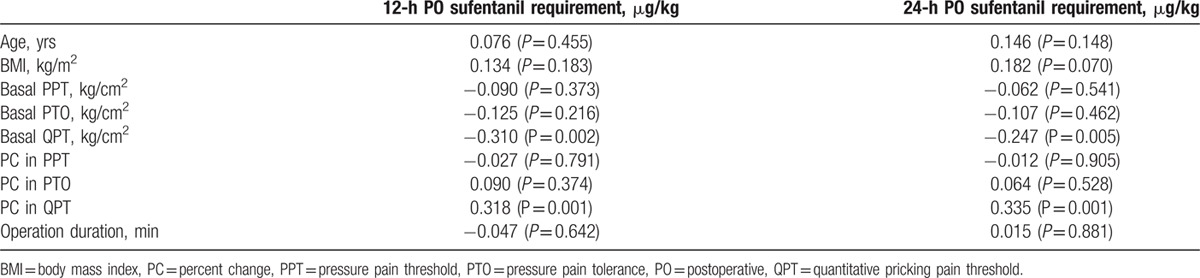
Pearson correlations between analgesic outcomes and preoperative data.

As shown in Table 4, 2 predictive factors (basal QPT and percent change in QPT) provided the best predictive model for sufentanil requirement within the first 12 hours after surgery (*r*^2^ = 0.138, *P* < 0.001). The percent change in QPT alone provided the best predictive model for total sufentanil requirement within the first 24 hours after surgery (*r*^2^ = 0.112, *P* = 0.001). Regression coefficients for the percent change in QPT for predicting12 and 24-hour sufentanil requirements were positive (0.226 and 0.335). These findings indicated that the percent change in QPT was an independent predictor, and that patients who were more sensitive to sufentanil required more sufentanil for postoperative pain control.

**Table 4:**
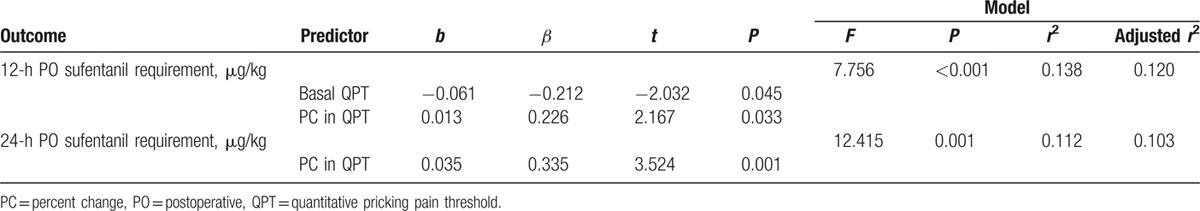
Multiple regression analyses of postoperative sufentanil requirements.

### 3.4 Exploratory study

**Figure 3:**
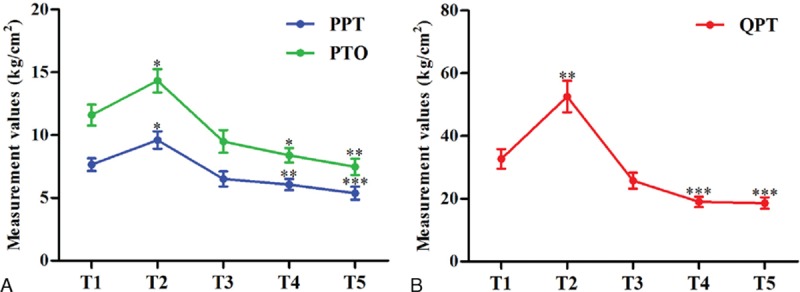
PPT, PTO, and QPT at different time points in the exploratory study. PPT = pressure pain threshold, PTO = pressure pain tolerance, QPT = quantitative pricking pain threshold. Compared with T1, ^∗^*P* < 0.05; ^∗∗^*P* < 0.01; ^∗∗∗^*P* < 0.001.

Measurements of PPT, PTO, and QPT at the anterior superior iliac spine are shown in Table 5. A repeated ANOVA revealed significant differences at the various predetermined time points. All values increased at T2 and decreased at T3 versus T1, but LSD testing of multiple comparisons showed that some of these changes were not significant (*P* > 0.05; Fig. 3). However, at T4 and T5, all values were significantly lower in comparison with T1 (Table 5; Fig. 3). Pearson correlation analysis results for preoperative and postoperative percent change in PPT, PTO, and QPT are listed in Table 6. The percent change in QPT at T3, T4, and T5 compared with T1 showed a strong positive correlation with that at T2 (*r*^2^ = 0.814, 0.811, and 0.794, respectively; *P* < 0.001 for all).

**Table 5:**
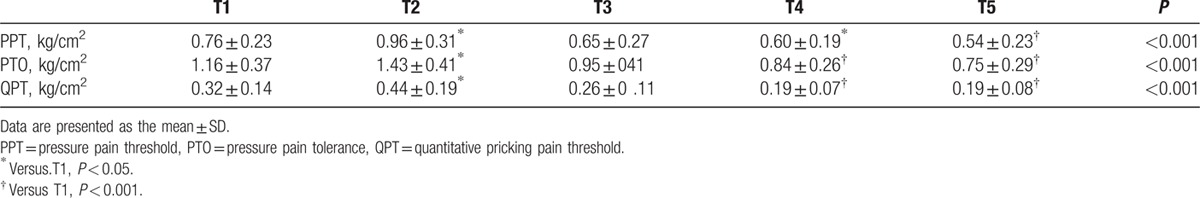
PPT, PTO, and QPT in the exploratory study.

**Table 6:**
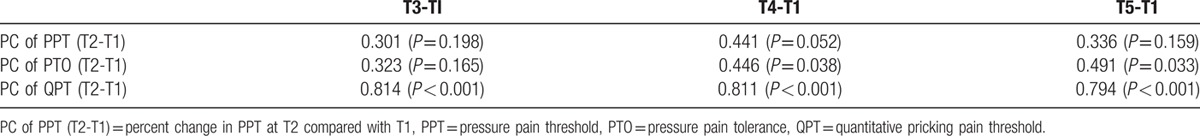
Pearson correlations between preoperative and postoperative measurements in the exploratory study.

### 3.5 Receiver-operating characteristic curve analysis in the primary study

**Figure 4:**
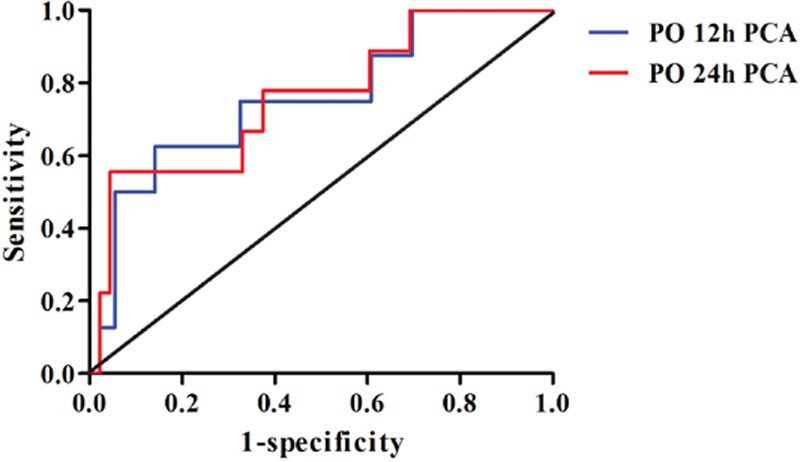
Receiver-operating characteristic curve for postoperative 12 and 24 hours PCA sufentanil requirement based on the percent change of quantitative pricking pain threshold in 100 patients in the exploratory study. PO = postoperative, PCA = patient-controlled analgesia.

The unilateral 90% normal reference for postoperative sufentanil requirement was calculated as 0.492 μg/kg at 12 hours and 0.938 μg/kg at 24 hours after surgery. We performed a ROC curve analysis to determine the optimal cut-off value for “excess requirement” based on the percent change in QPT. The areas under the ROC curves were 0.755 (95% confidence interval [CI] 0.573–0.938, progressive significance: *P* = 0.017) at 12 hours and 0.758 (95% CI 0.586–0.930, progressive significance: *P* = 0.011) at 24 hours after surgery (Fig. 4). The optimal cut-off values for 12 and 24-hour postoperative “excess requirement” was 188% based on the Youden index calculation, which yielded 0.446 (sensitivity 0.500 and specificity 0.946), and 0.512 (sensitivity 0.556 and specificity 0.956), respectively. This result indicated that patients with a percent change in QPT >188% had a >50% probability of requiring more sufentanil for postoperative pain control, whereas those with a percent change in QPT <188% were only approximately 5% more likely to require additional sufentanil for postoperative pain control.

## 4 Discussion

In this study, we determined individual sensitivity to sufentanil before induction of anesthesia in the operating room and investigated its ability to predict analgesic requirement for postoperative pain control. Experimental pain thresholds significantly increased after sufentanil infusion; unexpectedly, however, we observed a positive correlation between the percent change in QPT and postoperative sufentanil requirement. As this finding was counterintuitive, we performed a subsequent exploratory study to help explain this positive association.

Because experimental pain measurement^[26]^ and postoperative pain intensity are affected by sex,^[27]^ only female patients scheduled for gynecological surgery were recruited. Choosing this population also allowed us to eliminate artifacts arising from differences in surgical sites and methods, which may also affect postoperative pain intensity and analgesic requirements.^[28]^ In contrast to previous studies,^[29–31]^ we excluded patients who reported inadequate analgesia and required additional medications during the postoperative follow-up. Thus, data associated to postoperative sufentanil requirements for all patients were obtained under similar postoperative analgesic effects.

Since mechanical stimulation is more convenient and is associated with better patient acceptance than other forms of experimental stimulation,^[32]^ we selected pressure and pricking pain stimuli to evaluate the analgesic effect of sufentanil. Pain sensitivity to pressure, measured using a mechanical algometer with a 0.1-cm^2^ probe, is suitable for clinical applications, and pain sensitivity to pricking as a continuous variable can be measured using a 0.01-cm^2^ probe.^[18]^ In comparison with the conventional weighted method with a graded series of pinprick stimuli, the QPT test requires less time, obtains continuous data, and has a reduced risk of damage and infection, thus making it easier to apply in clinical settings. In the current study, we observed increased changes in PPT, PTO, and QPT 5 minutes after sufentanil infusion, indicating that these measures could successfully reflect the analgesic effect of sufentanil. Among the various mechanical pain measurements, only the percent change in QPT after sufentanil infusion was significantly correlated with postoperative sufentanil requirement, suggesting that important differences may exist between stimulation modalities. In this particular study, QPT possessed the highest predictive potential.

Our primary intuitive assumption was that a patient who was more sensitive to opioid analgesia would have a lower dose requirement for postoperative analgesia. However, in our study, patients with a higher percent change in QPT required more sufentanil (*β* = 0.318 and 0.335) for postoperative pain control, which was inconsistent with the intuitive assumption. This unexpected association prompted us to further investigate our findings. One of the primary causes of greater analgesic requirement is OIH, which may be induced by intraoperative remifentanil or other opioids.^[1,15,16,33]^ Thus, under the conditions of the primary study, we hypothesized that OIH might cause a correlation between preoperative sufentanil sensitivity and postoperative analgesic requirement.

Previous studies have shown the utility of quantitative sensory testing (QST) for the clinical assessment of OIH.^[22,23,25,34]^ We used this method to determine the occurrence of postoperative OIH, and also the correlation between OIH with preoperative sufentanil sensitivity. Our results showed that values for all types of experimental pain measures (PPT, PTO, and QPT) near the surgical incision were significantly lower after surgery than before, indicating increased pain sensitivity after surgery. Thus, OIH was a factor in the current study. We also found that the percent change in QPT after preoperative sufentanil infusion was positively associated with the percent change in QPT at postoperative time points, indicating that patients with a higher sensitivity to sufentanil were more likely to experience higher levels of postoperative OIH. Taken together, the results of our exploratory study supported our primary findings that patients with higher sensitivity to sufentanil had greater risk of requiring more sufentanil for postoperative pain control.

Our findings pose several potential advantages to opioid application in postoperative pain treatment. In the clinic, opioid analgesics are essential for anesthesia induction and postoperative pain control. In the present study, we used sufentanil sensitivity to predict sufentanil requirement without disrupting clinical routines or increasing costs. Additionally, our method can easily be applied to determine individual sensitivity to sufentanil before anesthesia induction in the operating room, as the assessment requires only a few minutes. Furthermore, we found that patients with higher sensitivity to sufentanil would need more sufentanil for postoperative pain treatment, as supported by the results of our secondary exploratory study. Most importantly, our ROC diagnostic analysis yielded an optimal cut-off value for predicting “excess” postoperative sufentanil requirement based on the percent change in QPT. The cut-off value for percent change in QPT (188%) may be useful as a marker for predicting increased postoperative sufentanil requirement and therefore could be used to facilitate the choice of more effective postoperative pain treatment strategies when using sufentanil. The preoperative predictive method and ROC diagnostic analysis might also be used as an effective tool for predicting the response to other opioid analgesics in postoperative pain control.

Several limitations should be considered when interpreting the results in the current study. First, to obtain a more accurate analysis of the association between the preoperative detection of sufentanil sensitivity and postoperative analgesic requirements, only female patients who received gynecologic surgery under general anesthesia were included in the current study. Thus, the results of the current study were based on a specific cohort, and further validation using another independent cohort is needed to strengthen these findings. Second, the ROC curve analysis was only built on data from the initial study; therefore it remains unclear whether the translation of the ROC cut-off value to other surgeries, populations, and anesthetics will be useful.

In conclusion, our findings demonstrate that preoperative detection of individual sensitivities to sufentanil by means of experimental pain measurements could predict postoperative analgesic requirements in patients undergoing gynecologic surgery. Further, our findings suggest that the percent change in QPT may serve as a marker for predicting postoperative sufentanil requirements in the future. Finally, the observed positive association, that is, “higher sensitivity but increased requirement,” may provide a novel viewpoint for good application of opioid analgesics in the perioperative period.
